# Recent advances in cohesin biology

**DOI:** 10.12688/f1000research.8881.1

**Published:** 2016-08-03

**Authors:** Susannah Rankin, Dean S. Dawson

**Affiliations:** 1 Program in Cell Cycle and Cancer Biology, Oklahoma Medicial Research Foundation, Oklahoma City, Oklahoma, USA; 2 Department of Cell Biology, University of Oklahoma Health Sciences Center, Oklahoma City, Oklahoma, USA

**Keywords:** cohesin, chromosome, mitosis

## Abstract

Sister chromatids are tethered together from the time they are formed in S-phase until they separate at anaphase. A protein complex called cohesin is responsible for holding the sister chromatids together and serves important roles in chromosome condensation, gene regulation, and the repair of DNA damage. Cohesin contains an open central pore and becomes topologically engaged with its DNA substrates. Entrapped DNA can be released either by the opening of a gate in the cohesin ring or by proteolytic cleavage of a component of the ring. This review summarizes recent research that provides important new insights into how DNA enters and exits the cohesin ring and how the rings behave on entrapped DNA molecules to provide functional cohesion.

## Introduction

**Figure 1.**
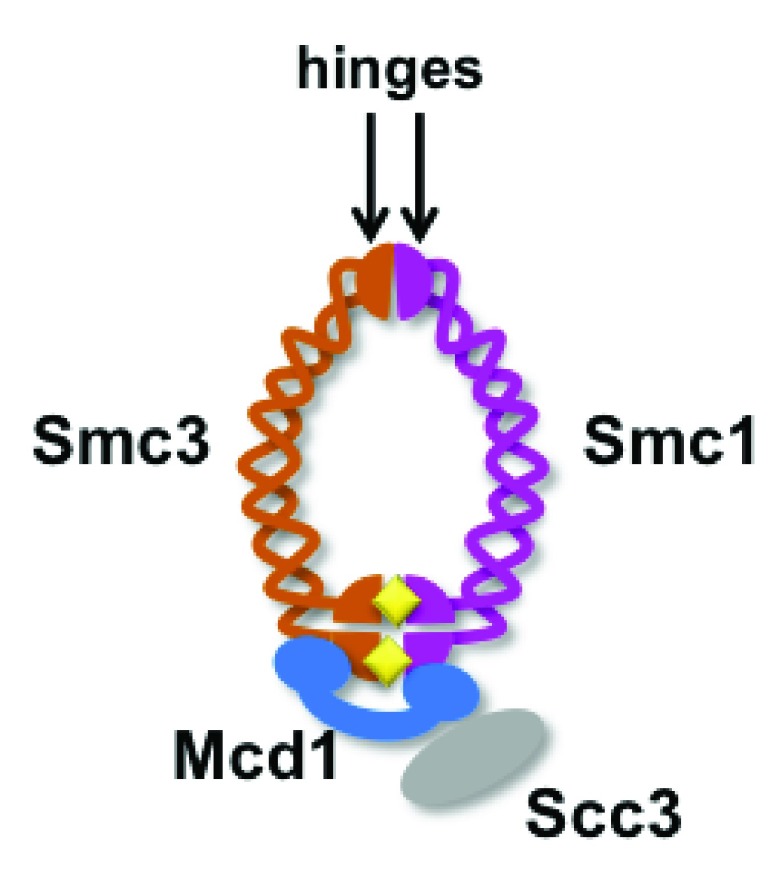
The cohesin complex. The four-subunit complex contains two SMC proteins, each of which forms long antiparallel coiled-coils. The SMC proteins interact in two places: where they fold back on themselves (hinge domains) and at head domains formed by interaction of their N and C termini. The interactions of the Smc1 and Smc3 head domains together form two intermolecular ATPases. ATP is indicated by yellow diamonds. Note that the Smc1 and 3 subunits, when interacting at both the hinge and the ATPase domains, would form a structure with an internal pore. The Mcd1 subunit (blue) interacts with both SMC head domains. Because the ATPase domains interact, a second pore may exist between Mcd1 and the Smc proteins. Separation of the Smc ATPase domains by ATP hydrolysis would create a single pore surrounded by Mcd1, Smc3, and Smc1. The Scc3 subunit, shown in grey, is not thought to form part of the ring.

Cohesin is a multi-subunit protein complex that tethers sister chromatids together each cell cycle, from S-phase until anaphase ( Figure 1). Cohesin allows the newly formed sister chromatids to attach to the mitotic spindle as a unit. By joining the spindle as a fixed pair, the sisters are able to form microtubule attachments that will ultimately pull them to opposite poles. The orderly and synchronous separation of the chromatids at anaphase is accomplished by the coordinated removal of cohesin from the chromatids.

Although cohesin was initially discovered in budding yeast, the subunits and the structure of the complex are highly conserved. Cohesin is composed of four structural components. Smc1 and Smc3 are members of the SMC family of proteins, which are involved in DNA organization in all organisms. These proteins fold over on themselves to form long, anti-parallel, coiled-coils. The “hinges” at one end of the proteins interact with each other, as do the head domains at the opposite end ( Figure 1). The head domains of each protein contain complementary partial ATPase domains that form two complete intermolecular ATPases when the heads interact. In mitotically growing cells, head domains are spanned by a subunit variously referred to as Mcd1, Scc1, or Rad21. The fourth subunit, Scc3, associates with Mcd1 but may also interact with the Smc1-Smc3 hinge domains ^1, 2^. Additional proteins regulate the behavior of cohesin: Wapl and Pds5 mediate both cohesion establishment and cohesin release, and in certain higher eukaryotes Sororin promotes the stable association of cohesin with the chromosomes (reviewed in 1– 3). The release of cohesin from chromosomes is inhibited by acetylation of Smc3 by members of the Eco1 family of acetyltransferases ^4– 7^.

Purified and reconstituted cohesin complexes can form a ring with a diameter of about 40 nm when observed by electron microscopy ^8– 10^, but whether this open form reflects its *in vivo* conformation is an important question. Cohesin holds sister chromatids together, at least in part, by topological entrapment of the DNA ^11, 12^. This leads to the question of how DNA enters and exits the cohesin pore. To determine which gates are the points of DNA entry into or exit from the ring, investigators have employed engineered versions of the cohesin proteins that form rings with gates that either are eliminated, by fusions of adjacent subunits, or can be artificially locked ^13, 14^. Collectively, these experiments suggest that chromatin can be tethered by cohesin when the Smc1-Mcd1 or Mcd1-Smc3 junctions are sealed, but not when the hinge-hinge junction is closed. But what triggers the junctions to open to allow chromatin entry or to open again when it is appropriate to release cohesin? The past two years have provided significant new insights into how the cohesin ring might be opening to allow the passage of DNA and how the ring might behave once it is topologically bound to chromatin. Here we briefly survey some of these exciting observations.

## Reversible loading and unloading of DNA into the cohesin ring

The synchronous separation of chromatids at anaphase is accomplished by cleavage of the Mcd1 subunit, but cohesin is also released from chromatin through a non-proteolytic process throughout the cell cycle and, in animal cells in particular, much of the cohesin is released from chromosomes in mitotic prophase. Structural studies have shown that the N-terminal end of Mcd1 interacts with the head proximal coiled-coil domain of Smc3 ^15^. Prior studies suggested that this interface acts as the chromatin exit gate; now this has been shown more directly ^16^. The authors used a system in which they could monitor the association of the N-terminal end of Mcd1 with Smc3 in living cells, using imaging and cross-linking studies. Elimination of Wapl ^Rad61^, or the presence of other mutations known to stabilize cohesin on chromosomes, had the effect of stabilizing the association of the Mcd1 N-terminus and Smc3. What triggers the release of the Mcd1-Smc3 interaction *in vivo*? A number of recent papers have focused on this question and provide compelling evidence that release requires ATP hydrolysis at the Smc1-Smc3 interface ^16– 19^. Most of these studies used genetic schemes that exploited the inviability of *eco1* mutants in budding yeast to identify mutations that stabilize cohesin (or reduce its release), thus restoring viability. These experiments revealed that mutations of the ATPase active site closest to the site at which Eco1 acetylates Smc3 could stabilize cohesin on DNA/chromatin, *in vitro* or *in vivo*, suggesting that cohesin release is promoted by this ATPase activity ^16– 19^. Interestingly, analogous mutations at the second ATPase site on the head domain could not stabilize cohesin ^17, 18^.

What is the relationship between the cohesin ATPases and the cohesion-destabilizing protein Wapl? Consistent with its role in cohesin removal, Wapl affects the ATPase-mediated release mechanism. Although Wapl does not stimulate the ATPase in an *in vitro* system, it does promote increased dissociation of DNA from the cohesin complex through release of the N-terminus of Mcd1 in an ATP-dependent manner ^19^. Indeed, fusion of Smc3 to the N-terminal end of Mcd1 renders cohesin removal from chromosomes Wapl resistant ^20^, and ectopic induction of Wapl expression *in vivo* triggers the dissociation of Smc3 and the N-terminus of Mcd1 ^16^.

Initial studies of the role of Wapl in chromosome cohesion revealed its cohesin unloading activity, but it was recently found to also promote the loading of cohesin onto DNA substrates *in vitro,* also through the Mcd1-Smc3 gate ^19^. The concept of DNA entering the cohesin ring through the Mcd1-Smc3 gate stands in contrast to previous work that suggested that opening of the Smc1-Smc3 hinge interface was critical for cohesin loading *in vivo*. This model was supported by experiments demonstrating that locking the hinge-hinge interface of engineered Smc1 and Smc3 proteins blocked cohesin loading ^13, 14^. Could Wapl promote cohesin loading through the Mcd1-Smc3 gate *in vivo*? This model is consistent with the observation that Wapl mutants show reduced overall cohesin loading ^21^ and is appealing for its simplicity in putting the regulation of entry/exit at a single portal. But in an apparent contradiction of this model, Wapl is not essential for sister chromatid cohesion in budding yeast ^4, 21^. Thus, if Wapl can indeed promote DNA entry, then there must be another way for chromatin to load (e.g. through the hinge interface). Alternatively, perhaps DNA entry can still occur through the Mcd1-Smc3 gate in Wapl mutants. This entry might be inefficient, or incomplete, but in a way that is still sufficient to promote cohesion. For example, DNA may be caught between Mcd1 and the Smc1-Smc3 heads, never entering the main pore. Additional studies of the way in which the cohesin ring interacts with and loads onto the DNA will be required to assess the precise role of Wapl in cohesion establishment.

**Figure 2.**
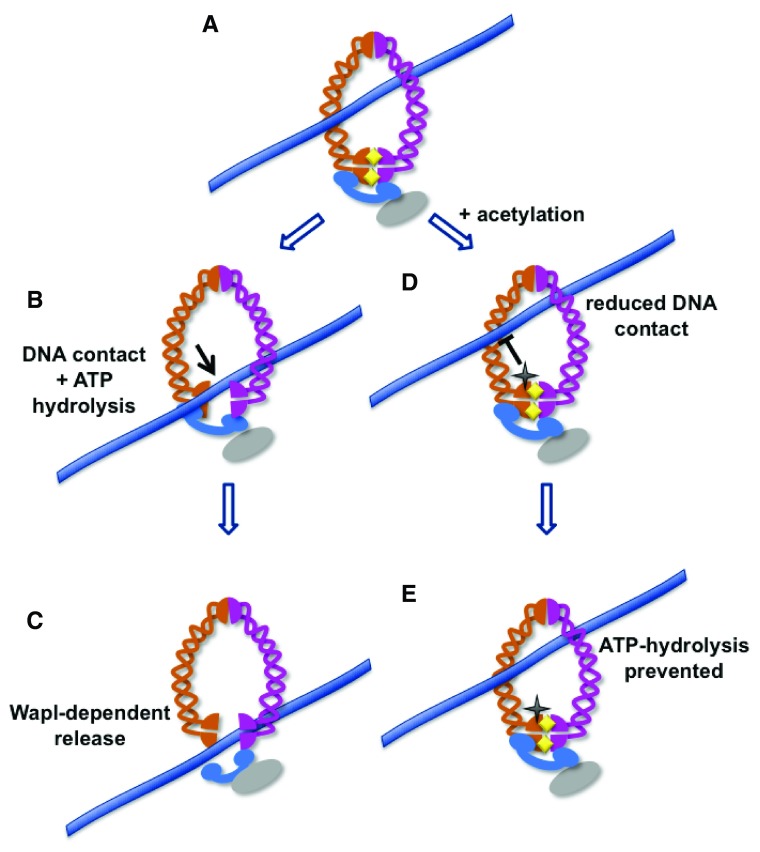
Model for DNA unloading through the Smc3-Mcd1 gate. DNA is loaded into the cohesin ring, which is closed in part through interaction of the Smc1 and Smc3 head domains ( **A**). Once loaded into the cohesin ring, interaction of DNA (lavender line) with the head domain of Smc3 can stimulate ATPase activity at the Smc3-Smc1 interface, resulting in partial opening of the cohesin ring ( **B**). This allows Wapl to open the Smc3-Rad21 interface, fully releasing the DNA ( **C**) ^19^. In contrast, acetylation of the Smc3 head domain, indicated by the black star, would prevent close interaction of the DNA with the head domains, preventing ATP hydrolysis ( **D**) and thus preventing ring opening ( **E**). In this model, acetylation of the Smc3 head domain might prevent both entry *and* exit of DNA.

What prevents the cohesin ring from disengaging from chromosomes prematurely once it is loaded? Acetylation of the Smc3 subunit of cohesin by Eco1 promotes cohesion and is thought to antagonize the releasing activity of Wapl. This is based on the observation that inviability caused by *ECO1* mutations can be suppressed by inactivation of Wapl ^5, 6, 21^. Consistent with this genetic observation, new *in vitro* studies indicate that Eco1 can block ATPase-dependent opening of the Mcd1-Smc3 gate ^19^. Interaction of cohesin with DNA stimulates ATPase activity, as if DNA alone might promote opening of the cohesin ring ^1, 18, 19^. Acetylation of Smc3 by Eco1 reduces this ATPase activity and thus stabilizes cohesin on DNA. These results have led to the model that direct interaction of DNA with Smc3 near the targets of acetylation activates the ATPase. Subsequent acetylation of Smc3 would then block interaction of Smc3 with the DNA, thereby preventing cohesin removal ( Figure 2). In fact, earlier work has shown that acetylation-mimicking mutations of Smc3 reduce *in vitro* interactions of cohesin with DNA and reduce loading of cohesin on chromosomes *in vivo* ^22^.

How is this DNA-dependent ATPase reaction entrained *in vivo*? Interaction of the cohesin loader complex, Scc2/4, with cohesin may function by stimulating a structural rearrangement of the cohesin ring, exposing the DNA binding region to promote loading and ATP hydrolysis ^1^. Future studies exploring the DNA-cohesin interaction and the ways in which Wapl, Pds5, and the loader complex affect ring conformations will be needed to further explore this model.

## Chromatid tethering by cohesin

**Figure 3.**
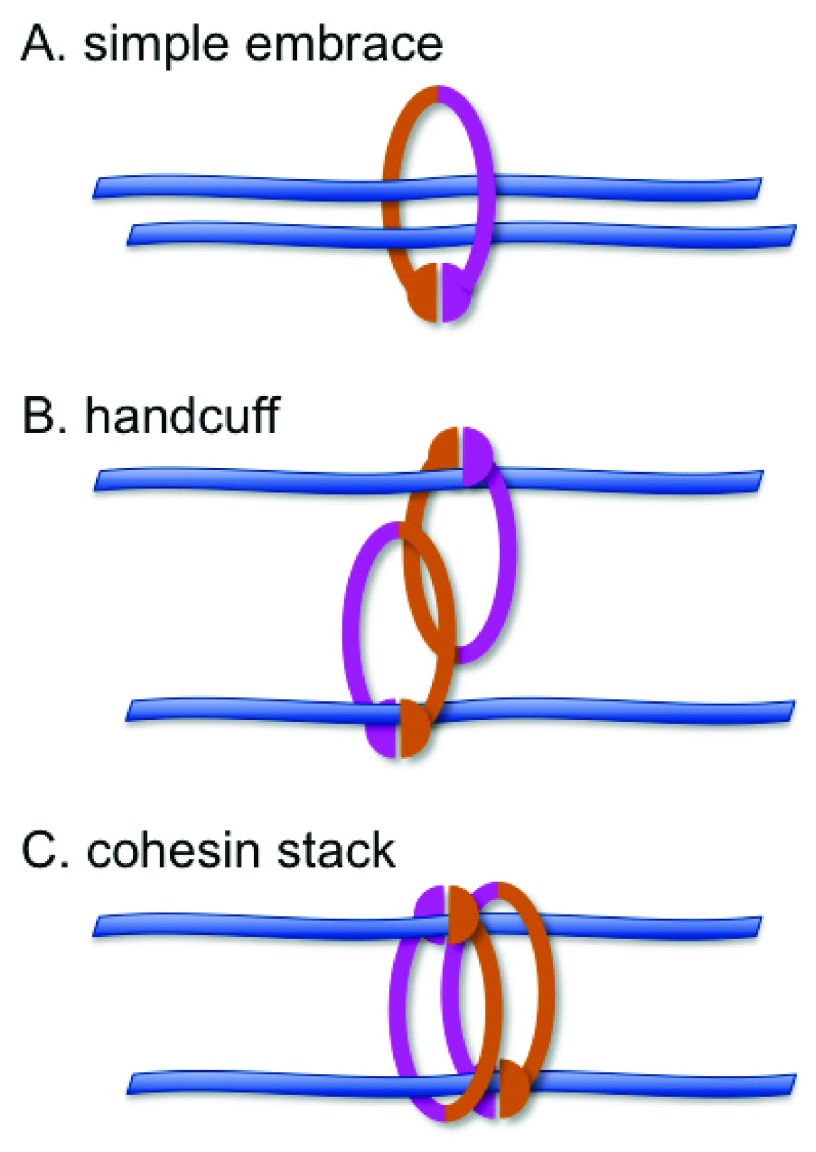
Different models for functional interaction of cohesin with sister chromatids. In the most basic “embrace” model ( **A**), both sister chromatids are entrapped together within individual cohesin rings, which are loaded either before or during DNA replication. The finding that two different non-functional alleles of individual cohesin subunits are able to promote cohesion when expressed in the same cell is consistent with the notion that higher order cohesin interactions can promote sister chromatid tethering ^26^. Examples include the handcuff ( **B**) and stacked cohesin ( **C**) models.

Once associated with chromosomes, how does cohesin tether sister chromatids together? The embrace model in its simplest form suggests that DNA from both sister chromatids become encircled by single cohesin rings coincident with their replication ( Figure 3) ^23^. In some models, this was suggested to occur by passage of the replication fork through the cohesin rings ^24^. Other models suggest that chromatids are encircled by separate cohesin rings (sometimes called handcuff models), which then oligomerize to tether the sisters together. None of these models are mutually exclusive, and cohesin might organize DNA through more than one mechanism. The question of how cohesin and chromatids are organized has been particularly difficult to study, as it has not yet been possible to visualize cohesin-DNA interactions in a native context. Biochemical isolation of cohesin has not provided evidence for high levels of oligomerization, but these approaches are challenged by the need to solubilize chromatin in order to purify the cohesin ^25^.

Recently, an indirect approach was used to provide evidence that cohesin rings might act as oligomers to mediate normal levels of cohesion ^26^. One prediction of an oligomerization model is that since multiple cohesin rings are acting together, in cells where each cohesin ring always has one of two different structural defects, rings with two different defects might be able to compensate for, or complement, one another by virtue of their combined contributions to a common oligomer ( Figure 3). Conversely, if the rings act independently, each could not supply what the other is missing. In this study, the investigators screened for the survival of cells when two different mutant forms of the same gene ( *MCD1* or *SMC3*) were expressed in the same cell. Remarkably, they identified pairs of mutant alleles that exhibited strong inter-allelic complementation. That is, the expression of either mutant allele alone in the cell led to loss of cohesion and inviability, while co-expression led to cohesion and viability. An oligomerization model is also supported by the finding that inactivation of Pds5 during mitosis leads to loss of cohesion, though cohesin proteins remain associated with the sister chromatids ^27, 28^.

Though indirect, the simplest explanation of these data is that cohesin rings can work together to fulfill their essential function. It may be that cohesin rings work either alone or as oligomers in wild-type cells, and in the examples of intra-allelic complementation, it is the ability of the rings to act as oligomers that allows the rescue of cohesion. The authors suggest revised models for ways in which oligomerization of rings could be part of the cohesion process and point out the flexibility that such oligomerization mechanisms might provide to cohesins in mediating long-range chromosomal interactions or responding to signals that require alterations in chromosome domain organization in order to facilitate appropriate gene expression.

## Observing interactions of cohesin with DNA

**Figure 4.**
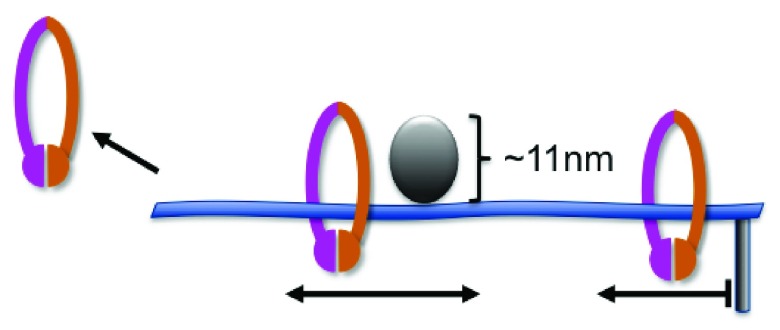
Single molecule analysis of cohesin’s interaction with DNA. Purified cohesin incubated with tethered and extended DNA molecules shows the behaviors illustrated ^29^. The presence of the loader complex (not shown) results in the highly stable interaction of cohesin with DNA. Once loaded, cohesin appears to move freely along the length of the DNA, unless there is an obstacle of greater than ~11 nm in size. Cohesin is released from the untethered end of the DNA molecule (shown at left) and stops when it reaches the tethered end (right).

Several cohesion models predict that the cohesin complex should be able to slide along the DNA and, based on the size of the central pore *in vivo*, suggest that cohesin should be able to pass over obstacles along the chromatin. In particular, passage of the replisome through the cohesin ring could explain how pairs of sister chromatids become encircled by single cohesin molecules. An elegant study has recently addressed properties of cohesin on chromatin ^29^. Using single molecule analysis, Stigler *et al*. assessed the binding and mobility of purified cohesin on defined DNA curtains (linear arrays of ordered DNA molecules of defined sequence) using total internal reflection fluorescence microscopy ( Figure 4). First, they showed that although cohesin alone has the intrinsic ability to interact with DNA, the loader complex is required for the efficient generation of very stably bound, salt-resistant complexes. This population presumably represents the topologically engaged cohesin, and it can be seen to translocate along the DNA molecules at a rate approaching the theoretical free diffusion rate, consistent with the idea of topological engagement around an open pore. Consistent with this model, the authors also showed that stably bound cohesin could slide off DNA molecules at non-tethered ends, much like a curtain ring sliding off a free curtain rod. They further showed that the cohesin complex could be pushed along DNA strands by motor proteins. Finally, they tested the pore size of the cohesin ring by determining the size limit of the obstacles that the ring can bypass during translocation. This was accomplished by engineering different sized obstacles to engage in a site-specific manner on the DNA curtain. They conclude that the pore size is in fact far smaller than that predicted based on electron microscopy studies and can accommodate obstacles of only ~11 nm, the approximate size of a nucleosome. Based on these analyses, the intrinsically crowded nature of most chromatin is predicted to greatly reduce, though not block, the diffusion of cohesin over distances of several kilobases in biologically relevant time frames. These findings fit well with the model that cohesin rings can be pushed to the ends of open reading frames by the transcriptional apparatus ^30, 31^ but raise questions as to whether the replisome, which is estimated to be closer to 20 nm ^32^, can pass through the cohesin ring.

## Summary

It remains to be seen how the physical properties of loaded cohesin, as revealed by this recent collection of groundbreaking experiments, are entrained to result in the tethering of sister chromatids in a robust manner. Does some small fraction of cohesin entrap two DNA molecules in much the same manner as seen in the single molecule analyses? Or do higher order interactions between cohesin complexes ensure inter-sister tethering, as suggested by the genetic studies? Whatever the answers, we are certainly much closer to understanding the nature of the interaction between chromosomes and cohesin than we were before these papers provided new, key insights into the nature of cohesin-DNA interactions.

## References

[1] Murayama Y Uhlmann F: Biochemical reconstitution of topological DNA binding by the cohesin ring. *Nature.* 2014;505(483):367–71. 10.1038/nature12867 24291789 PMC3907785

[2] Mc Intyre J Muller EG Weitzer S: *In vivo* analysis of cohesin architecture using FRET in the budding yeast *Saccharomyces cerevisiae*. *EMBO J.* 2007;26(16):3783–93. 10.1038/sj.emboj.7601793 17660750 PMC1952217

[3] Peters J Nishiyama T: Sister chromatid cohesion. *Cold Spring Harb Perspect Biol.* 2012;4(11): pii: a011130. 10.1101/cshperspect.a011130 23043155 PMC3536341

[4] Rowland BD Roig MB Nishino T: Building sister chromatid cohesion: smc3 acetylation counteracts an antiestablishment activity. *Mol Cell.* 2009;33(6):763–74. 10.1016/j.molcel.2009.02.028 19328069

[5] Rolef Ben-Shahar T Heeger S Lehane C: Eco1-dependent cohesin acetylation during establishment of sister chromatid cohesion. *Science.* 2008;321(5888):563–6. 10.1126/science.1157774 18653893

[6] Unal E Heidinger-Pauli JM Kim W: A molecular determinant for the establishment of sister chromatid cohesion. *Science.* 2008;321(5888):566–9. 10.1126/science.1157880 18653894

[7] Zhang J Shi X Li Y: Acetylation of Smc3 by Eco1 is required for S phase sister chromatid cohesion in both human and yeast. *Mol Cell.* 2008;31(1):143–51. 10.1016/j.molcel.2008.06.006 18614053

[8] Gruber S Haering CH Nasmyth K: Chromosomal cohesin forms a ring. *Cell.* 2003;112(6):765–77. 10.1016/S0092-8674(03)00162-4 12654244

[9] Anderson DE Losada A Erickson HP: Condensin and cohesin display different arm conformations with characteristic hinge angles. *J Cell Biol.* 2002;156(3):419–24. 10.1083/jcb.200111002 11815634 PMC2173330

[10] Huis in 't Veld PJ Herzog F Ladurner R: Characterization of a DNA exit gate in the human cohesin ring. *Science.* 2014;346(6212):968–72. 10.1126/science.1256904 25414306

[11] Ivanov D Nasmyth K: A physical assay for sister chromatid cohesion *in vitro*. *Mol Cell.* 2007;27(2):300–10. 10.1016/j.molcel.2007.07.002 17643378

[12] Haering CH Farcas AM Arumugam P: The cohesin ring concatenates sister DNA molecules. *Nature.* 2008;454(7202):297–301. 10.1038/nature07098 18596691

[13] Buheitel J Stemmann O: Prophase pathway-dependent removal of cohesin from human chromosomes requires opening of the Smc3-Scc1 gate. *EMBO J.* 2013;32(5):666–76. 10.1038/emboj.2013.7 23361318 PMC3590994

[14] Gruber S Arumugam P Katou Y: Evidence that loading of cohesin onto chromosomes involves opening of its SMC hinge. *Cell.* 2006;127(3):523–37. 10.1016/j.cell.2006.08.048 17081975

[15] Gligoris TG Scheinost JC Bürmann F: Closing the cohesin ring: structure and function of its Smc3-kleisin interface. *Science.* 2014;346(6212):963–7. 10.1126/science.1256917 25414305 PMC4300515

[16] Beckouët F Srinivasan M Roig MB: Releasing Activity Disengages Cohesin's Smc3/Scc1 Interface in a Process Blocked by Acetylation. *Mol Cell.* 2016;61(4):563–74. 10.1016/j.molcel.2016.01.026 26895425 PMC4769318

[17] Elbatsh AM Haarhuis JH Petela N: Cohesin Releases DNA through Asymmetric ATPase-Driven Ring Opening. *Mol Cell.* 2016;61(4):575–88. 10.1016/j.molcel.2016.01.025 26895426 PMC4769319

[18] Camdere G Guacci V Stricklin J: The ATPases of cohesin interface with regulators to modulate cohesin-mediated DNA tethering. *Elife.* 2015;4: pii: e11315. 10.7554/eLife.11315 26583750 PMC4709263

[19] Murayama Y Uhlmann F: DNA Entry into and Exit out of the Cohesin Ring by an Interlocking Gate Mechanism. *Cell.* 2015;163(7):1628–40. 10.1016/j.cell.2015.11.030 26687354 PMC4701713

[20] Chan KL Roig MB Hu B: Cohesin's DNA exit gate is distinct from its entrance gate and is regulated by acetylation. *Cell.* 2012;150(5):961–74. 10.1016/j.cell.2012.07.028 22901742 PMC3485559

[21] Sutani T Kawaguchi T Kanno R: Budding yeast Wpl1(Rad61)-Pds5 complex counteracts sister chromatid cohesion-establishing reaction. *Curr Biol.* 2009;19(6):492–7. 10.1016/j.cub.2009.01.062 19268589

[22] Hu B Itoh T Mishra A: ATP hydrolysis is required for relocating cohesin from sites occupied by its Scc2/4 loading complex. *Curr Biol.* 2011;21(1):12–24. 10.1016/j.cub.2010.12.004 21185190 PMC4763544

[23] Nasmyth K Haering CH: Cohesin: its roles and mechanisms. *Annu Rev Genet.* 2009;43:525–58. 10.1146/annurev-genet-102108-134233 19886810

[24] Haering CH Lowe J Hochwagen A: Molecular architecture of SMC proteins and the yeast cohesin complex. *Mol Cell.* 2002;9(4):773–88. 10.1016/S1097-2765(02)00515-4 11983169

[25] Zhang N Kuznetsov SG Sharan SK: A handcuff model for the cohesin complex. *J Cell Biol.* 2008;183(6):1019–31. 10.1083/jcb.200801157 19075111 PMC2600748

[26] Eng T Guacci V Koshland D: Interallelic complementation provides functional evidence for cohesin-cohesin interactions on DNA. *Mol Biol Cell.* 2015;26(23):4224–35. 10.1091/mbc.E15-06-0331 26378250 PMC4642856

[27] Kulemzina I Schumacher MR Verma V: Cohesin rings devoid of Scc3 and Pds5 maintain their stable association with the DNA. *PLoS Genet.* 2012;8(8):e1002856. 10.1371/journal.pgen.1002856 22912589 PMC3415457

[28] Tong K Skibbens RV: Cohesin without cohesion: a novel role for Pds5 in *Saccharomyces cerevisiae*. *PLoS One.* 2014;9(6):e100470. 10.1371/journal.pone.0100470 24963665 PMC4070927

[29] Stigler J Camdere GÖ Koshland DE: Single-Molecule Imaging Reveals a Collapsed Conformational State for DNA-Bound Cohesin. *Cell Rep.* 2016;15(5):988–98. 10.1016/j.celrep.2016.04.003 27117417 PMC4856582

[30] Lengronne A Katou Y Mori S: Cohesin relocation from sites of chromosomal loading to places of convergent transcription. *Nature.* 2004;430(6999):573–8. 10.1038/nature02742 15229615 PMC2610358

[31] Bausch C Noone S Henry JM: Transcription alters chromosomal locations of cohesin in *Saccharomyces cerevisiae*. *Mol Cell Biol.* 2007;27(24):8522–32. 10.1128/MCB.01007-07 17923700 PMC2169412

[32] Sun J Shi Y Georgescu RE: The architecture of a eukaryotic replisome. *Nat Struct Mol Biol.* 2015;22(12):976–82. 10.1038/nsmb.3113 26524492 PMC4849863

